# Modifying effects of education on the association between lifestyle behaviors and the risk of obesity: evidence from South Korea

**DOI:** 10.1186/s12889-016-3776-4

**Published:** 2016-10-20

**Authors:** Woojin Chung, Sunmi Lee, Seung-ji Lim, Jaeyeun Kim

**Affiliations:** 1 Department of Health Policy, Graduate School of Public Health, Institute of Health Services Research, Yonsei University, 50-1 Yonsei-ro, Seodaemun-gu, Seoul, 03722 Republic of Korea; 2 Health Insurance Policy Research Institute, National Health Insurance Service, 32 Geongang-ro, Wonju-si, Gangwon-do 26464 Republic of Korea; 3 Department of Public Health, Graduate School, Yonsei University, 50-1 Yonsei-ro, Seodaemun-gu, Seoul 03722 Republic of Korea

**Keywords:** Obesity, Education, Lifestyle behavior, Interaction, Sex, South Korea

## Abstract

**Background:**

No previous study has explored the interactions between education and lifestyle in relation to obesity. This study hypothesized that education may be obesogenic through its interplay with lifestyle behaviors.

**Methods:**

Data for a nationally representative sample (6937 men and 9333 women) from the Korea National Health and Nutrition Examination Survey (2010–2012) were analyzed. Multivariate logistic regressions were performed for three education levels and six lifestyle behaviors, each of which comprised two groups.

**Results:**

Interactions between education and lifestyle behaviors in relation to obesity were observed for all lifestyle behaviors in women (*p* for interaction <0.001) and for three lifestyle behaviors in men. Education appeared obesogenic for three groups of lifestyle behaviors in men (*p* for trend <0.05), but was protective against obesity for 11 groups in women. Each one-unit increase in education level in men increased the odds of obesity by 1.29-fold among under-reported energy intake groups (95 % confidence interval: 1.16, 1.44).

**Conclusions:**

Education may be a risk factor for obesity through its interplay with lifestyle behaviors. Further research is required to examine these findings in different socio-cultural settings.

**Electronic supplementary material:**

The online version of this article (doi:10.1186/s12889-016-3776-4) contains supplementary material, which is available to authorized users.

## Background

Obesity is becoming a serious global health issue. Obesity can lead to premature death due to coronary heart disease, high cholesterol, hypertension, diabetes, gallbladder disease, or asthma, in addition to a decreased quality of life [1]. On a societal level, obesity can cause increased medical spending as well as decreased productivity in labor markets [2].

A number of studies, conducted from both academic and policy perspectives, have attempted to identify factors related to increased risk of obesity. Among them, many studies have paid special attention to the importance of lifestyle behaviors in relation to obesity [3–8]. Lifestyle behaviors commonly recognized to favor health have been shown to reduce obesity risk [9]. Other studies have emphasized the role of increased education in lowering obesity risk. In general, higher levels of education are thought to have a protective effect against obesity [10, 11]. However, the other study have suggested that greater educational attainment have a obesogenic effect against obesity [12].

Unfortunately, no study to date has performed a multi-dimensional analysis of the associations between lifestyle behaviors and education in relation to obesity. This lack leaves studies of obesity far behind those of other health outcomes or of risk factors in exploring the interplay between or among various influencing factors [13, 14], and may also be a barrier to developing theories and designing appropriate policies for reducing obesity.

This study analyzed data for a nationally representative South Korean adult sample aged over 25 years, who had most likely completed their education, in order to investigate the interaction between education level and six types of lifestyle behaviors, i.e., smoking, drinking alcohol, physical exercise activity, daily sleep duration, daily energy intake, and level of stress in relation to obesity. This study postulated that education level might modify the association between lifestyle behaviors and obesity. It was also hypothesized that through its interplays with lifestyle behaviors, education might exert a protective effect against obesity in regard to certain lifestyle behaviors, but might also have an obesogenic effect in regard to other lifestyle behaviors.

## Methods

### Study population

This study used data from the Fifth Korea National Health and Nutrition Examination Survey (KNHANES V, 2010–2012), conducted by the Korea Centers for Disease Control and Prevention [15]. Representative data on the non-institutionalized, general population of South Korea were collected through a stratified, multistage probability sampling design. To assure an equal probability of being sampled, weightings were assigned to each respondent.

Of 25,534 (8958, 8518, and 8058 in 2010, 2011, and 2012, respectively) randomly selected individuals, 24,173 were interviewed. Of these, 17,534 individuals aged 25 years or more were chosen for this study because they had most likely already completed their education [10]. Finally, this study analyzed 16,270 (92.8 %) participants (6937 men and 9333 women) with complete information.

### Measures

The body mass index for every participant was calculated based on height and body weight measured during the physical examination. According to the guidelines proposed by the World Health Organization indicating that Asians have a lower average body mass index [16], this study defined obesity as a body mass index of 25 or higher.

Education level, defined as the highest level of formal education completed as of the interview date, comprised three levels: junior high school or less (≤9 years of education), senior high school (10–12 years of education), and college or higher (≥ 13 years of education). This study examined six lifestyle behaviors: smoking, drinking alcohol, physical exercise activity, daily sleep duration, daily energy intake, and level of stress. Each lifestyle behavior was categorized into two groups as follows: current smoking status (smoking or non-smoking); risk from drinking alcohol (no or low risk, or, medium or higher risk, according to the sex-specific guidelines of the World Health Organization for risk of acute problems from drinking alcohol) [17]; physical exercise activity (active or inactive, where inactive was defined as the lack of participation in either moderate-intensity physical exercise activity for at least 30 min at a time per day for at least 5 days per week or vigorous-intensity physical exercise activity for at least 20 min at a time per day for at least 3 days per week [18]; daily sleep duration (short or long, according to whether a participant sleeps for 7 or more hours per day); daily energy intake (under-reported or not under-reported); and the level of stress (stressful or not stressful). Thus, a total of 12 groups of lifestyle behaviors were considered. Daily energy intake was obtained from 24-hour dietary recalls and a 63-item, open-ended food frequency questionnaire composed of foods that were regarded as key sources of energy for our population [19]. Energy intake was categorized as “under-reported” if it was lower than the estimated energy requirement obtained from the Institute of Medicine’s predictive equations [20, 21].

The analysis incorporated a variety of potential confounders: sex (male or female), age (25–44, 45–54, 55–64, or 65 years and older), marital status (married or non-married), residential area (urban or rural), employment status (employed or unemployed), and income (below median income, or median income or higher). Non-married included never married, separated, widowed, or divorced. An equivalized household income was used to adjust for household size. A chronic disease variable was included for participants with hypertension, dyslipidemia, and diabetes mellitus at the time of the survey. Variables indicating survey year were included to control for time-fixed effects. For each multivariate model focusing on the association of education level and each lifestyle behavior with obesity, the remaining lifestyle behaviors were added as potential confounders.

### Statistical analysis

Each lifestyle behavior for each sex was compared across education levels using *χ* ^2^ tests. The estimated prevalence of obesity for each group of a lifestyle behavior was compared across education levels for each sex, using *χ* ^2^ tests.

Before examining the hypothesis that education level modifies the association between lifestyle behaviors and obesity, this study first examined whether the association between each lifestyle behavior and obesity differed between men and women by constructing a simple logistic regression model with an interaction term between sex and each lifestyle behavior in relation to obesity. Because the *p*-values for all of the interaction terms were highly significant (*p* for interaction <0.001), the remaining analyses were performed separately for each sex.

This study then examined the hypothesis using both unadjusted and adjusted models. For the former, the interaction terms between education and each lifestyle behavior were fitted in the logistic regression models without any potential confounders. For the latter, the logistic regression models were adjusted for all studied confounders, where potential multicollinearity between independent variables was not a concern because the variance inflation factors ranged between 1.0 and 3.6.

The adjusted odds ratios (ORs) of obesity and 95 % confidence intervals (CIs) were estimated for the interaction terms between education and each lifestyle behavior. The significance of the interaction terms were examined separately for each combination of lifestyle behavior groups and education level, and jointly for all combinations. In the case of significance jointly considering all combinations, the null hypothesis was that the interaction terms in the regression models were all equal to zero. In doing so, this study could test whether education level modified the association between each lifestyle behavior and obesity after adjusting for all studied confounders.

In addition, using the education variable in its continuous form ranging from 1 to 3 (junior high school or less = 1, senior high school = 2, and college or higher = 3), the education trend effect for each lifestyle behavior in relation to obesity was estimated separately for each group of lifestyle behaviors, after adjusting for all studied confounders. In examining the significance of the interaction terms between education and each group of lifestyle behavior, this study could test whether education level obesogenically or protectively modified the association between each group of lifestyle behaviors and obesity. Finally, the interactions were fully illustrated on a graph, plotting the ORs of obesity calculated for each combination of education level and a group of lifestyle behaviors relative to their referent (Additional file 1).

All analyses were conducted considering the complex survey design, and the statistical significance was set at an alpha level of 0.05. To examine the significance of interaction terms, this study used the Wald test to adjust for the design effect, rather than a likelihood ratio test, because estimation procedures of logistic regression adjusting for the design effect use quasi-likelihoods rather than true likelihoods. Data were analyzed using SAS 9.2 (SAS Institute, Cary, NC, USA) and STATA 12 software (StataCorp, College Station, TX, USA).

## Results

### Participant characteristics and obesity prevalence

The overall prevalence of obesity was 37.5 % (standard error, 0.8) in men and 30.2 % (0.7) in women with a significant difference between sexes (*p* < .001). Table 1 shows that the participant characteristics were differentially distributed across education levels for each sex, except for sleep duration (*p* = .50), energy intake (*p* = .86) and survey year (*p* = .66) in men and survey year (*p* = .54) in women.

**Table 1:** Distribution (%) of study sample characteristics according to educational level and sex: Korea National Health and Nutrition Examination Survey, 2010–2012

Characteristics	Men (*N* = 6937)				Women (*N* = 9333)			
	Junior high school or less	Senior high school	College or higher	*p*	Junior high school or less	Senior high school	College or higher	*p*
Age, year				<.001				<.001
25–44	3.3	36.5	57.0		1.9	47.1	75.0	
45–54	15.4	24.6	22.1		17.1	32.8	17.9	
55–64	29.5	20.5	10.9		30.2	13.4	5.4	
65 or over	51.8	18.4	10.0		50.8	6.7	1.8	
Non-married	10.3	17.0	19.4	<.001	35.2	15.6	23.8	<.001
Rural	36.7	20.2	11.1	<.001	32.9	14.1	8.9	<.001
Employed	64.9	78.0	84.0	<.001	43.5	48.8	51.8	<.001
Median or higher income	27.8	53.2	71.3	<.001	28.9	56.9	73.7	<.001
Smoking status				<.001				<.001
Non-smoking	63.8	54.9	59.0		95.4	93.0	96.1	
Smoking	36.2	45.1	41.0		4.6	7.0	3.9	
Risk from drinking alcohol				<.001				<.001
No or low	68.8	46.5	43.3		85.6	67.5	67.6	
Medium or higher	31.2	53.5	56.8		14.4	32.5	32.4	
Physical exercise				<.01				<.01
Active	17.6	23.4	20.8		16.0	18.6	14.1	
Inactive	82.4	76.6	79.3		84.0	81.4	85.9	
Sleep duration				.50				<.001
Long	57.8	59.3	57.0		48.2	61.6	67.2	
Short	42.2	40.7	43.0		51.8	38.4	32.8	
Energy intake				.86				.01
Not under-reported	72.7	74.2	75.4		84.0	82.9	84.8	
Under-reported	27.3	25.8	24.6		16.1	17.1	15.2	
Stress				<.001				.01
Not stressful	82.6	78.3	71.1		72.5	73.0	69.4	
Stressful	17.4	21.7	28.9		27.6	27.0	30.6	
Self-rated health, poor	26.9	12.7	10.6	<.001	35.1	15.1	11.3	<.001
Chronic disease	42.4	28.0	18.1	<.001	50.1	16.5	5.4	<.001
Survey year				.66				.54
2010	35.9	34.8	35.8		33.7	33.7	34.4	
2011	34.2	34.0	33.7		34.5	34.4	33.7	
2012	30.0	31.3	30.6		31.8	31.9	31.9	
All participants	31.0	32.7	36.3		44.8	29.5	25.7	

For the sake of brevity, the descriptive statistics were shown as % and unweighted

*P*-values were obtained by the *χ*2 test considering the complex survey design

*N* number, *Obesity* body mass index ≥25

Table 2 shows that the obesity prevalence differed across education levels for each sex. In addition, the obesity prevalence for each group of lifestyle behaviors varied across education levels except for no- or low-risk from drinking alcohol (*p* = .05), active physical exercise (*p* = .41), under-reporting of energy intake (*p* = .48) and being stressed (*p* = .06) in men and smoking (*p* = .15) in women.

**Table 2:** Obesity prevalence rate (%) by educational level and sex: Korea National Health and Nutrition Examination Survey, 2010–2012

Characteristics	Men (*N* = 6937)							Women (*N* = 9333)						
	Junior high school or less		Senior high school		College or more		*p*	Junior high school or less		Senior high school		College or more		*p*
	Rate	(SE)	Rate	(SE)	Rate	(SE)		Rate	(SE)	Rate	(SE)	Rate	(SE)	
All participants	31.9	(1.3)	38.0	(1.3)	40.5	(1.3)	<.001	39.7	(1.0)	30.0	(1.2)	17.1	(1.0)	<.001
Smoking status							<.001*							<.001*
Non-smoking	34.3	(1.6)	36.4	(1.8)	40.5	(1.7)	.04	40.3	(1.0)	29.5	(1.2)	16.9	(1.0)	<.001
Smoking	28.4	(2.1)	36.6	(1.8)	40.5	(2.0)	<.001	30.5	(3.7)	34.6	(4.3)	21.4	(5.2)	.15
Risk from drinking alcohol							<.001*							<.001*
No or low risk	29.7	(1.6)	31.3	(1.7)	35.5	(1.9)	.05	38.6	(1.1)	29.9	(1.4)	18.1	(1.2)	<.001
Medium or higher risk	35.2	(2.3)	42.2	(1.7)	43.7	(1.7)	.02	44.7	(2.5)	30.1	(2.1)	15.4	(1.6)	<.001
Physical exercise							<.001*							<.001*
Active	34.1	(2.8)	38.6	(2.8)	39.5	(2.6)	.41	41.6	(2.4)	32.6	(2.5)	25.0	(3.4)	<.001
Inactive	31.4	(1.5)	37.8	(1.5)	40.8	(1.4)	<.001	39.3	(1.1)	29.4	(1.3)	15.8	(1.0)	<.001
Sleep duration							<.001*							<.001*
Long	31.2	(1.7)	38.0	(1.5)	39.0	(1.7)	.01	39.6	(1.3)	28.5	(1.4)	15.2	(1.2)	<.001
Short	32.8	(2.0)	37.9	(2.0)	42.6	(1.8)	<.01	39.8	(1.4)	32.4	(1.8)	21.1	(1.8)	<.001
Energy intake							<.001*							<.001*
Not under-reported	26.8	(1.5)	33.7	(1.5)	37.2	(1.5)	<.001	36.1	(1.1)	26.0	(1.2)	14.8	(1.0)	<.001
Under-reported	45.2	(2.7)	49.3	(2.6)	49.6	(2.4)	.48	56.9	(2.5)	46.6	(2.9)	29.6	(3.0)	<.001
Stress							<.001*							<.001*
Not stressful	31.7	(1.4)	36.7	(1.4)	39.9	(1.6)	<.001	39.1	(1.2)	28.5	(1.3)	16.7	(1.2)	<.001
Stressful	32.6	(3.2)	42.1	(2.7)	41.9	(2.2)	.06	41.1	(1.8)	33.5	(2.2)	18.0	(1.8)	<.001

All analyses were conducted considering the complex survey design

*P*-values were obtained by the *χ*2 test

*N* number, *SE* standard error, *Obesity* body mass index ≥25

*P-values were obtained by the Wald test for the interaction terms between education and each lifestyle behavior fitted in the logistic regression models without any potential confounders

### Interaction between education and each lifestyle behavior in relation to obesity

Table 2 shows the results of the Wald tests of interaction terms between education and each lifestyle behavior in relation to obesity, without adjusting for potential confounders. The interaction terms were highly significant both in men and women (*p* for interaction < .001), which suggests that, before adjustment, education level modified the association between each lifestyle behavior and obesity both in men and in women.

The results after adjustments are shown in Table 3 for men and Table 4 for women. In men, interaction terms between education and each lifestyle behavior in relation to obesity were jointly significant for smoking status (*p* for interaction = .01), risk from drinking alcohol (*p* for interaction < .001), and energy intake (*p* for interaction < .001). In contrast, education in women interacted significantly with all six lifestyle behaviors in relation to obesity (*p* for interaction < .001).

**Table 3:** Interaction effect of education and each lifestyle behavior on obesity in men (*N* = 6937): Korea National Health and Nutrition Examination Survey, 2010–2012

Characteristics	Junior high school or less		Senior high school		College or higher		*p* for interaction	Education trend for each group		
	OR	(95 % CI)	OR	(95 % CI)	OR	(95 % CI)		OR	(95 % CI)	*p* for trend
Smoking status							.01			
Non-smoking	1.00		1.01	(0.80–1.28)	1.02	(0.81–1.30)		1.12	(1.01–1.24)	.03
Smoking	0.66	(0.50–0.86)	0.79	(0.62–1.01)	0.93	(0.71–1.22)		1.05	(0.94–1.17)	.41
Risk from drinking alcohol							<.001			
No or low risk	1.00		0.93	(0.73–1.18)	1.08	(0.84–1.39)		1.00	(0.89–1.11)	.95
Medium or higher risk	1.19	(0.90–1.56)	1.50	(1.18-1.90)	1.56	(1.21–2.01)		1.16	(1.05–1.29)	<.01
Physical exercise							.76			
Inactive	1.00		1.10	(0.89–1.35)	1.20	(0.95–1.51)		1.09	(0.98–1.20)	.11
Active	1.04	(0.77–1.41)	1.08	(0.81–1.46)	1.14	(0.86–1.52)		1.07	(0.96-1.20)	.21
Sleep duration							.33			
Long	1.00		1.11	(0.89–1.38)	1.13	(0.88–1.46)		1.06	(0.96–1.18)	.24
Short	1.08	(0.84–1.38)	1.14	(0.88–1.47)	1.34	(1.03–1.73)		1.11	(1.00–1.24)	.05
Energy intake							<.001			
Not under- reported	1.00		1.14	(0.91–1.44)	1.28	(1.02–1.62)		1.01	(0.91–1.12)	.89
Under- reported	2.29	(1.74–3.02)	2.23	(1.71–2.92)	2.19	(1.65–2.91)		1.29	(1.16–1.44)	<.001
Stress							.41			
Not stressful	1.00		1.03	(0.84–1.26)	1.16	(0.93–1.45)		1.08	(0.97–1.19)	.17
Stressful	0.96	(0.68–1.34)	1.26	(0.95–1.68)	1.22	(0.94–1.59)		1.12	(1.00–1.24)	.05

All analyses were conducted considering the complex survey design

*P* value was obtained by the Wald test

All estimates for all the interaction terms between education each health behavior were obtained from the multivariate logistic regression models, adjusted for age, marital status, residential area, employment status, equivalized household income, self-rated health, chronic disease, survey year, and the other lifestyle behaviors

*N* number, *OR* odds ratio, *CI* confidence interval, *Obesity* body mass index ≥25

**Table 4:** Interaction effect of education and each lifestyle behavior on obesity in women (*N* = 9333): Korea National Health and Nutrition Examination Survey, 2010–2012

Characteristics	Junior high school or less		Senior high school		College or higher		*p* for interaction	Education trend for each group		
	OR	(95 % CI)	OR	(95 % CI)	OR	(95 % CI)		OR	(95 % CI)	*p* for trend
Smoking status							<.001			
Non-smoking	1.00		0.81	(0.67–0.97)	0.49	(0.39–0.62)		0.72	(0.64–0.80)	<.001
Smoking	0.68	(0.47–0.98)	1.14	(0.75–1.73)	0.67	(0.35–1.29)		0.79	(0.66–0.94)	.01
Risk from drinking alcohol							<.001			
No or low risk	1.00		0.91	(0.75–1.11)	0.59	(0.47–0.76)		0.71	(0.64–0.80)	<.001
Medium or higher risk	1.37	(1.09–1.71)	0.99	(0.77–1.28)	0.50	(0.36–0.70)		0.72	(0.64–0.81)	<.001
Physical exercise							<.001			
Inactive	1.00		0.86	(0.71–1.04)	0.48	(0.38–0.60)		0.70	(0.63–0.78)	<.001
Active	1.12	(0.89–1.41)	0.98	(0.75–1.28)	0.82	(0.55–1.22)		0.80	(0.70–0.91)	<.01
Sleep duration							<.001			
Long	1.00		0.80	(0.64–0.99)	0.44	(0.34–0.58)		0.69	(0.62–0.77)	<.001
Short	1.00	(0.86–1.15)	0.92	(0.74–1.15)	0.63	(0.48–0.82)		0.75	(0.67–0.84)	<.001
Energy intake							<.001			
Not under- reported	1.00		0.83	(0.69–1.01)	0.51	(0.40–0.65)		0.65	(0.58–0.73)	<.001
Under- reported	2.25	(1.79–2.82)	2.18	(1.63–2.90)	1.23	(0.88–1.71)		0.99	(0.87–1.12)	.85
Stress							<.001			
Not stressful	1.00		0.81	(0.67–0.98)	0.50	(0.39–0.64)		0.71	(0.63–0.79)	<.001
Stressful	0.96	(0.80–1.15)	0.97	(0.75–1.25)	0.54	(0.40–0.73)		0.74	(0.65–0.83)	<.001

All analyses were conducted considering the complex survey design

*P* value was obtained by the Wald test

All estimates for all the interaction terms between education each health behavior were obtained from the multivariate logistic regression models, adjusted for age, marital status, residential area, employment status, equivalized household income, self-rated health, chronic disease, survey year, and the other lifestyle behaviors

*N* number, *OR* odds ratio, *CI* confidence interval, *Obesity* body mass index ≥25

### Education trend effect for each lifestyle behavior: protective or obesogenic?

Analysis of the interaction terms between education level and each lifestyle behavior, where education level was in its continuous form, are shown in Table 3 for men and Table 4 for women. In men, interaction terms with the continuous form of education level were significantly positive in three of the 12 groups of lifestyle behaviors (non-smoking, medium or higher risk from drinking alcohol, and under-reporting of energy intake). For each one-unit increase education level, the odds of obesity increased by 1.29-fold in the group with under-reported energy intake (95 % CI: 1.16, 1.44) and by 1.12-fold in the non-smoking group (95 % CI: 1.01, 1.24). These findings suggest that education may be obesogenic in men through its interplay with a certain group of lifestyle behaviors, including non-smoking, medium or higher risk from drinking alcohol, and under-reporting of energy intake.

In contrast, except under-reporting of energy intake, the interaction terms between the continuous form of education level and each group of lifestyle behaviors in women were significantly negative. For example, for each one-unit increase in education level, the odds of obesity diminished by 0.69-fold in those who slept for 7 h or more per night (95 % CI: 0.62, 0.77) and by 0.71-fold in those with no or low risk from drinking alcohol (95 % CI: 0.64, 0.80). These findings suggest that education appears to have a protective effect against obesity in women through its interplay with every group of lifestyle behaviors, except for under-reporting of energy intake.

## Discussion

The findings of this study suggest that education level may modify the association between lifestyle behaviors and obesity in a nationally representative sample of adults in South Korea. Before adjustment, models revealed that education level was an effect modifier in both men and women for all lifestyle behaviors. In adjusted models, the results differed between men and women. In men, education level was an effect modifier for three lifestyle behaviors: smoking status, risk from drinking alcohol, and energy intake. In contrast, in women, the modifying effects of education were observed in each of the six lifestyle behaviors assessed in this study. Based on these findings, it appears that the modifying effects of education level on the associations between lifestyle behaviors and obesity depends on both sex and lifestyle behavior.

This study also reports that education may have an obesogenic, protective, or no effect at all on obesity, depending on sex and lifestyle behavior. In men, education appears to have an obesogenic effect through its interplay with three groups of lifestyle behaviors: non-smoking, medium or higher risk from drinking alcohol, and under-reporting of energy intake. However, education appears to have no effect on obesity in the remaining nine groups of lifestyle behaviors. In contrast, education appears to have a protective effect against obesity in women, through its interplay with 11 of the 12 groups of lifestyle behaviors. However, education had no effect on obesity through its interplay with a group of under-reporting of energy intake.

### Comparisons to previous studies

Previous studies of associations of lifestyle behaviors and education level with obesity have mainly focused on main effects [22, 23]. With respect to the relationship between lifestyle behaviors and obesity, many studies suggest that individual lifestyles may influence obesity risk, in a way that unhealthy lifestyle behaviors increase obesity risk, though the effects may differ by sex. These lifestyle behaviors include smoking [4, 24]; drinking alcohol [5, 25]; leisure-time physical activity [6, 26]; sleep duration [7, 27]; energy intake [8, 28]; and psychosocial stress [3, 29].

A great deal of attention has been paid to the relationship between education level and obesity. These studies could be categorized into several groups according to a level of socioeconomic development and sex [22, 23]. In societies with high levels of socioeconomic development, the relationship between education and obesity in men has been mostly reported as nonsignificant; however, this relationship in women has been observed as mostly negative, according to a study performed in Luxembourg [11]. In societies with medium levels of development, the relationship is largely nonsignificant or positive in men, but mainly negative in women [30, 31]. Finally, in societies with low levels of development, the relationship appears to be positive in both men and women [32].

To date, no studies of obesity have assessed the modifying effect of education on the associations between lifestyle behaviors and obesity. Previous studies have examined the role of education as an effect modifier in the association of wealth or occupation with obesity only in women in developing countries. For example, analysis of a sample of women of reproductive age showed that education protected against the obesogenic effect of wealth in Egypt [33]. Another study examined the interaction between education and wealth on obesity in women aged 15–49 years in low- and middle-income countries [34]. While no effect was observed in three countries (India, Nigeria, and Benin), an interaction was found in four countries (Colombia, Peru, Jordan, and Egypt). In these countries, wealth was associated with increased obesity risk in women with primary education or less, whereas it either decreased or did not influence obesity risk in women with higher levels of education.

Another study of women aged 60 years or more in four provinces in China provided evidence of the interaction between education and occupation on abdominal obesity. In women with no education, the odds of abdominal obesity in the sedentary occupation group were more than double compared to those of the agricultural occupation group, despite the lack of evidence for such a relationship among women with any education [35].

### Plausible mechanisms

Several potential reasons may explain the finding that education level may modify the association between lifestyle behaviors and obesity. First, it is possible that a forward causal link exists between or among three variables; i.e., education level, lifestyle behavior, and obesity, such that higher levels of education predict a healthy lifestyle behavior, which in turn predicts reduced obesity [36]. Second, there might be a reverse causal link such that being obese may cause an unhealthy lifestyle behavior, thereby resulting in a lower education level [10]. Third, notwithstanding the three variables, a fourth variable such as peer group, taste, and genetics might be associated with at least one of these three variables. Fourth, an individual may seek not only to have better health but also to consume more goods for personal enjoyment [36]. For example, if a lifestyle behavior, which tends to increase obesity risk, allows an individual to earn more, and thus consume more goods, the individual might prefer such lifestyle behaviors to others that may protect against obesity. Finally, higher education may lead to increased knowledge on nutrition, which has been previously shown to be associated with lower body mass index and lower rates of obesity [37, 38]. Moreover, knowledge on nutrition could explain the observed sex differences, as women may be more health conscious than men, irrespective of their education. This is especially true in regard to certain unhealthy behaviors, such as overeating and smoking [39, 40].

If we consider these five potential reasons collectively, a cross-sectional, two-way association between education level, a lifestyle behavior, or obesity may reflect a complex set of multi-faceted interactions between or among education level, lifestyle behaviors, obesity, and various fourth variables. As a result, depending on demographic group, time, and society, education may be obesogenic, be protective against obesity, or exert no effect at all, in the association between lifestyle behaviors and obesity. In particular, sex-based differences in the modifying effect of education may derive from sex-based differences in unobserved fourth variables. In countries undergoing rapid socioeconomic transitions, such as China, India, and South Korea [41], cultural or religious norms are likely to impose higher costs for obesity on women than on men in both the labor and marriage markets [42, 43].

### Public health implications

To the best of our knowledge, this is the first study to report a multi-dimensional analysis of the associations between education and lifestyle behaviors in relation to obesity. Though caution is necessary when making policy suggestions based on findings from cross-sectional data, the findings of the current study suggest that increased education may reduce, increase, or have no effect on obesity risk based on the interplay with various lifestyle behaviors, depending on sex.

Increased education in women could protect against obesity through its interplays with most lifestyle behaviors except for under-reporting of energy intake, whereas increased education in men could be obesogenic through its interplays with non-smoking, medium or higher risk from drinking alcohol, and under-reporting of energy intake. This study offers empirical evidence to support studies that have cautioned against unintended consequences of obesity reduction programs through enhanced education [44].

### Strengths and limitations

This study assessed data from sample of nationally representative South Korean adults. The sample provided abundant information about anthropometric measures, socio-demographic status, lifestyle behaviors, psychological factors, and diagnosed diseases. The sample also provided detailed information on energy intake. Above all, this study is the first to address the modifying effects of education on the associations between various lifestyle behaviors and obesity.

This study has several limitations. The cross-sectional study design does not allow causal inference to be made about the relationships among education level, lifestyle behaviors, and obesity. Moreover, this study may be subject to a selection bias due to the choice of education level. Some information based on self-reporting could lead to measurement errors and recall bias. Certain characteristics, such as social network, parental obesity status, tastes, genetics, and diet quality, were omitted because of data limitations.

## Conclusion

In summary, depending on lifestyle behaviors and sex, education level may modify the association between lifestyle behaviors and the risk of obesity. In South Korea, education appears to offer protection against obesity in women through its interplay with most lifestyle behaviors except for under-reporting of energy intake. In contrast, education appears to work obesogenically in men through its interplay with certain lifestyle behaviors. This is the first study to explore the importance of interactions between education level and lifestyle behaviors in relation to obesity. Future research on increasing adiposity levels should consider complex interactions between or among education level, lifestyle behaviors, obesity, and various fourth variables as well as examine the differences in these interactions according to a level of socioeconomic development and sex.

## Additional file

Additional file 1: Figure S1. Interaction effect of education and each lifestyle behavior on obesity in men and in women: Korea National Health and Nutrition Examination Survey, 2010–12 (DOCX 81 kb)

## Abbreviations

CIs: 95 % confidence intervals
ORs: Adjusted odds ratios
