# Supplementary material for: Modifying effects of education on the association between lifestyle behaviors and the risk of obesity: evidence from South Korea
Source: BMC Public Health. 2016 Oct 20;16:1100. doi: 10.1186/s12889-016-3776-4 (PMC5072334; doi:10.1186/s12889-016-3776-4)
Supplement: Additional file 1: Figure S1. — Interaction effect of education and each lifestyle behavior on obesity in men and in women: Korea National Health and Nutrition Examination Survey, 2010–12 (DOCX 81 kb) [file 12889_2016_3776_MOESM1_ESM.docx]

**Figure** Interaction effect of education and each lifestyle behavior on obesity in men and in women: Korea National Health and Nutrition Examination Survey, 2010-12

Each point represents the odds ratio (and its 95% confidence intervals) of that combinations of education level and each lifestyle behavior with the reference category indicating the unit odds ratio. All estimates for all the interaction terms between education each health behavior were obtained from the multivariate logistic regression models, adjusted for age, marital status, residential area, employment status, equivalized household income, self-rated health, chronic disease, survey year, and the other lifestyle behaviors.
